# Gut Microbiome Composition Linked to Inflammatory Factors and Cognitive Functions in First-Episode, Drug-Naive Major Depressive Disorder Patients

**DOI:** 10.3389/fnins.2021.800764

**Published:** 2022-01-28

**Authors:** Penghong Liu, Mingxue Gao, Zhifen Liu, Yanyan Zhang, Hongwei Tu, Lei Lei, Peiyi Wu, Aixia Zhang, Chunxia Yang, Gaizhi Li, Ning Sun, Kerang Zhang

**Affiliations:** ^1^First Hospital of Shanxi Medical University, Taiyuan, China; ^2^Department of Psychiatry, First Hospital of Shanxi Medical University, Taiyuan, China

**Keywords:** major depressive disorder, gut microbiome, diversity, inflammatory factors, cognitive function

## Abstract

**Objective:**

The microbiota–gut–brain axis, especially the inflammatory pathway, may play a critical role in the pathogenesis of cognitive impairment in major depressive disorder (MDD). However, studies on the microbiota-inflammatory-cognitive function axis in MDD are lacking. The aim of the present study was to analyze the gut microbiota composition and explore the correlation between gut microbiota and inflammatory factors, cognitive function in MDD patients.

**Method:**

Study participants included 66 first-episode, drug naïve MDD patients as well as 43 healthy subjects (HCs). The composition of fecal microbiota was evaluated using16S rRNA sequencing and bioinformatics analysis. The cytokines such as hs-CRP, IL-1β, IL-6, IL-10, and TNF-α in peripheral blood were detected *via* enzyme linked immunosorbent assay (ELISA); assessment of cognitive functions was performed using the Color Trail Test (CTT), The Repeatable Battery for the Assessment of Neuropsychological Status (RBANS) and the Stroop Color-Word Test (SCWT).

**Results:**

We found that compared with HCs, MDD patients had cognitive impairments and showed different α-diversity and β-diversity of gut microbiota composition. LDA effect size (LEfSe) analysis found MDD have higher *Deinococcaceae* and lower *Bacteroidaceae, Turicibacteraceae, Clostridiaceae* and *Barnesiellaceae* at family level. *Deinococcus* and *Odoribacter* was higher in the MDD group, however, *Bacteroides, Alistipes, Turicibacter, Clostridium, Roseburia*, and *Enterobacter* were lower at genus level. Furthermore, In MDD patients, the *Bacteroidaceae* and *Bacteroides* were both positively correlated with hsCRP, CCT1, CCT2. *Alistipes* was positively correlated with IL-6, Word time, Color time, Word-Color time, Color-Word time and negatively correlated with Delayed Memory, Total score and Standardized score. *Turicibacteraceae* and *Turicibacter* were both negatively correlated with IL-1β and IL-6.

**Conclusion:**

The present findings confirm that the gut microbiota in MDD patients have altered gut microbes that are closely associated with inflammatory factors and cognitive function in MDD patients.

## Introduction

Gut microbiota functions as a vital actor in the bidirectional communication between the digestive system and the central nervous system (CNS), also called the brain–gut–microbiota axis (Rhee et al., 2009). Increasingly, gut microbiota appears to influence brain functions through the autoimmune and endocrinology pathways (Huang et al., 2019). In recent decades, researchers have demonstrated the presence of bidirectional communication between the gut and brain *via* the brain–gut–microbiota axis (Yong et al., 2019; Yang et al., 2020; Liu et al., 2021). Some cross-sectional studies have found altered gut microbes in individuals with mental disorders (Dickerson et al., 2017; Bruce-Keller et al., 2018), particularly in major depressive disorder (MDD) (Sanada et al., 2020).

A study conducted by Naseribafrouei et al. (2014) analyzed the state of gut microbiota in individuals with MDD and healthy controls (HCs), and reported that when Bacteroidales, *Oscillibacter*, and *Alistipes* were present in higher levels, the *Lachnospiraceae* were linked with depressive symptoms. Other researchers studied the gut microbiota present in active-MDD (A-MDD) subjects, responding-MDD (R-MDD) subjects, and HCs (Jiang et al., 2015). They discovered that MDD patients had increased Proteobacteria, Bacteroidetes, and Actinobacteria, as well as decreased *Faecalibacterium*. Additionally, *Faecalibacterium* was negatively associated with depressive symptom severity (Jiang et al., 2015). Zheng et al. (2016) found higher levels of Actinobacteria and Bacteroidetes and lower levels of Firmicutes in MDD patients; they also found that transferring MDD feces into germ-free (GF) mice resulted in depressive phenotypes in recipient mice.

Gut microbiota plays a significant role in the development of the gut’s immune system, and host–microbe interactions impact the gut’s immunological homeostasis (Furusawa et al., 2013). When gut microbiota is disturbed, microbial-associated molecular patterns—those such as lipopolysaccharide (LPS) and bacterial lipoprotein—can activate immune cells and toll-like receptors (TLR) to trigger the release of pro-inflammatory cytokines. The effect is to increase the permeability of the gut–blood barrier and blood–brain barrier (BBB), and to regulate the function and behavior of the CNS (Petra et al., 2015). As such, gut microbiota may affect CNS function by inducing an inflammatory response. In addition, the vagus nerve (VN) can connect the CNS to the enteric nervous system (ENS), which can directly facilitate immunoregulatory signals to the brain and gut (Breit et al., 2018).

Cognitive deficits are present in patients with neuropsychiatric disorders. Impaired cognitive function has been described in the MDD patients. Numerous studies, reviews, and meta-analyses show a considerable impairment in inhibition, shifting, verbal working memory, visuospatial working memory, and verbal fluency (Ahern and Semkovska, 2016). Gut microbiota is closely related to cognitive function. Studies conducted using animal models have uncovered mutual interaction between the brain and gut microbiota in the development of Alzheimer’s disease (AD). It has been found that GF mice showed non-spatial and working memory impairment when studied alongside specific pathogen free (SPF) mice (Gareau et al., 2011). Desbonnet et al. (2015) found that antibiotic treatment significantly reduced the abundance and diversity of intestinal microbiota in weaned mice, and that non-spatial memory also declined on the Novel Object Recognition Test (NORT). Clinical trials have confirmed the presence of increased *Escherichia*, *Shigella*, *Bacteroides*, and *Ruminococcus*, in addition to the decreased *E*. *rectale*, *Bifidobacterium*, and *Dialister* in the feces of the cognitively impaired elderly, or in AD patients (Cattaneo et al., 2017; Vogt et al., 2017). After undergoing fecal microbiota transplantation from a healthy donor, AD patients displayed swift improvement in both memory and mood (Hazan, 2020). These studies locate a causal relationship between gut microbial composition and mental cognition (Chen et al., 2021). However, the mechanism by which gut microbiota affects cognitive function remains unclear. Some scholars have proposed inflammation may be responsible for the cognitive impairment of several chronic diseases, such as depression and insomnia (Strawbridge et al., 2015; Lamers et al., 2019). Depression has been associated with an increase in cytokines secretion (Zou et al., 2018), which can also lead to cognitive impairment affecting attention, learning, and memory (Misiak et al., 2018).

In conclusion, gut microbiota may participate significantly in the occurrence and development of cognitive dysfunction. However, previous studies on the relationship between gut microbes and cognitive function have focused on dementia patients or mouse models, and few studies have explored the connection in MDD patients. In light of these findings and the limitations of previous studies, we hypothesize that compared with HCs, changes in the gut microbiota could be seen in the first-episode untreated MDD group. Furthermore, dysbiosis of gut microbiota may be related to inflammatory factors and cognitive function in MDD patients.

## Materials and Methods

### Participants

A total of 66 first-episode, drug-naive MDD subjects between 18 and 55 years of age were taken from the inpatient and outpatient units of the First Hospital of Shanxi Medical University Department of Psychiatry during the period of December 2019 to July 2021. Each patient’s symptom was in accordance with the criteria for MDD presented by the Diagnostic and Statistical Manual of Mental Disorders-Fourth Edition (DSM-IV). We used HAMD-17 to measure the severity of depression, and scores of 17 or higher were accepted for the study (Hamilton, 1960). In order to screen for manic or hypomanic episodes, or for other psychiatric disorders, we used the Mini International Neuropsychiatric Interview (MINI) (Sheehan et al., 1998). Subjects found with the following conditions were excluded from the study: (1) autoimmune diseases, heart diseases, hepatobiliary and gastrointestinal diseases, blood diseases, diabetes, neurological diseases, mental retardation, or other psychiatric diseases; (2) pregnancy or lactation; (3) treatment in the last month with antibiotics or anti-inflammatory agents; (4) or had taken probiotics in the past 2 months. To recruit HCs, advertisements were used throughout the local community, with a total of 43 HCs gathered. HCs underwent the same exclusion criteria as MDD subjects. Participants from both groups submitted written informed consent to participate. This research project was approved by the Research Ethics Review Board of First Hospital of Shanxi Medical University in Taiyuan, China.

### Cognitive Function Assessment

We implemented the Polish version of the Color Trail Test (CTT) created by Łojek and Stańczak (Tyburski et al., 2020) to evaluate participants’ cognitive flexibility. The CTT is a culture-free form of the Trail Making Test (Reichenberg, 2010) and is made up of two parts: (a) CTT 1, during which the participants are asked to connect a series of 25 numbered circles randomly printed on paper, and (b) CTT 2, during which the participants are asked to connect circles numbered from 1 to 25, and that alternate in color between pink and yellow.

The Repeatable Battery for the Assessment of Neuropsychological Status (RBANS) (Randolph et al., 1998) is frequently implemented to assess cognitive function in cases of dementia, and it consists of 12 subtests. These measure five indices: Immediate Memory, Visuospatial/Constructional, Language, Attention, and Delayed Memory. The indices are scored using a composite score; as such, this study analyzes subtest scores as well as composite scores. To measure the battery, individual subtest scores were converted into age-corrected index scores, and a sum of the index scores was valued as the total score.

The Stroop Color-Word Test (SCWT) was used to measure inhibitory functions (Stroop, 1935). We used the classical version of the SCWT to evaluate cognitive inhibition (dominant verbal response; Stroop, 1935). In the first task (word), a patient is asked to rapidly read color words aloud (e.g., green, blue, yellow, red); the colors are printed in black ink on a standard white A4 sheet. The second task (color) requires the patient to verbally identify the colors of rectangles printed on a standard white A4 sheet. For the third task (word-color), the patient is asked to quickly read aloud the color words printed in mismatched colors (e.g., text reading “yellow” but written in red). In the fourth task (color-word), the patient must quickly name the color in which each word is printed, ignoring its word information. Researchers verbally expressed to the patients the instructions before each of the tasks. Based on Stroop (1935), the naming time was scored in all tests.

### Collection of Fecal and Blood Samples

After patients completed the SCWT, we collected stool samples (2 g) and immediately froze them at −80°C for the gut microbiota assay. To identify gut microbial communities, we implemented the 16S ribosomal RNA (16S rRNA) gene sequence-based approach. We collected 10 ml of blood using normal aseptic techniques, and we separated the plasma using centrifugation at 3500 × *g* at 4°C for 10 min. We stored it at −80°C to measure inflammatory parameters, including hs-CRP, IL-1β, IL-6, IL-10, and TNF-α *via* enzyme-linked immunosorbent assay (ELISA).

### Fecal Sample Collection, DNA Extraction, 16S rRNA Gene Sequencing, and Bioinformatics Data Analysis

The collection of fecal DNA was implemented by the use of the QIAamp^®^ DNA Stool Mini Kit (Qiagen, Hilden, Germany), following the maker’s instructions. By utilizing a NanoDrop One spectrophotometer (Thermo Fisher Scientific, Fitchburg, WI, United States), we quantified the DNA and then measured the integrity and size using 0.8% agarose gel electrophoresis. In order to amplify the 16S rRNA gene of bacteria, we utilized isolated DNA following the 338F universal primers (5′-ACTCCTACGGG AGGCAGCA-3′) and 806R (5′-GGACTACHVGGGTWTCTAAT-3′), focusing on the hypervariable regions V3-V4 of bacterial 16S rRNA. The Illumina Novaseq PE250 platform by Personal Biotechnology, Co., Ltd. was used to conduct PCR amplification and preparation of sequencing library (Shanghai, China).

The open-source software QIIME 2 (version: 2019.1) was used to process the 16S rRNA raw sequencing data (Bolyen et al., 2019). We demultiplexed the sequences and removed the V3/V4 primers with cutadapt (v2.8). We also implemented the DADA2 plugin package for the amplicon workflow: quality filtering, sequence truncation, denoise (error correction), sample inference, merging of paired-end reads, chimera identification and removal, singletons removal, and dereplication of sequences into amplicon sequence variants (ASV) with 100% sequence similarity (Callahan et al., 2016). Any OTUs found to have a frequency of <0.1% of the total number of reads were excluded. A Naive-Bayes classifier trained against the SILVA 132 database (Quast et al., 2013) was used to perform taxonomic classification, targeting the V3/V4 region of the 16S rRNA. Using QIIME 2 (version: 2019.1), we calculated the alpha and beta diversity metrics. Alpha diversity indices of the Abundance-based Shannon index, Simpson index, Observed Species index, Chao index, Faith’s PD index, Pielou’s evenness index, and Good’s coverage index were taken based on rarefied sequence count. An unsupervised principal coordinates analysis (PCoA) of Jaccard dissimilarity was used to conduct beta diversity analysis in order to identify variances of microbiome composition profiles at the ASV level. Linear discriminant analysis effect size (LEfSe) analysis (Segata et al., 2011) was implemented to locate distinguishing taxa in MDD and HCs at multiple levels, and to conceptualize the results in a cladogram and bar plot. We used Spearman’s correlation coefficient to create the correlation matrix among microbiome composition in addition to cytokines and cognitive function. The online genescloud platform^1^ was used to generate a heat map of the correlation matrix. To find which gut microbiota could act as biomarkers for discriminating between MDD patients and HCs at the genus/ASV level, we applied a Random Forest (RF) and Boruta machine learning algorithm (Speiser et al., 2019), and the region below the receiver operating characteristic curve (AUC) was utilized to measure the classification performance.

### Statistical Analyses

All of the analyses carried out in this study were undertaken using IBM SPSS Statistics Version 23.0 (SPSS-23). Two-sample *t*-tests were conducted to uncover distinctions in age, total years of education, total HAMD-17 scores, and the cognitive function scores of participants. *X*^2^ was used to estimate group differences in gender. The threshold of statistical significance was *P* < 0.05 (two-tailed). To determine the correlation coefficient between the gut microbiota and inflammatory parameters and clinical symptoms, either Pearson or Spearman correlation analyses were implemented.

## Results

### Clinical Characteristics and Inflammatory Parameters

We uncovered no significant discrepancies in the age, gender, or BMI of the MDD patients and HCs (*P* > 0.05). Patients with MDD had significantly fewer years of education than HCs (*P* < 0.001). The MDD group had higher total HAMD-17 scores than the HC group (*P* < 0.001). MDD patients spent significantly longer at the CCT1 and CCT2 stages than HCs (*P* < 0.05). In RBANS, healthy subjects scored significantly higher on Immediate Memory, Language, Attention, Delayed Memory, Total score, and Standardized score than MDD patients (*P* < 0.05). The score of Visuospatial/Constructional was not significantly different between the two groups (*P* > 0.05). In the Stroop Color/Word task, the MDD group showed longer Word time, Color time, and Color-Word time (*P* < 0.05); however, we uncovered no significant difference between the groups’ Word-Color times (*P* > 0.05).

We detected higher levels of hs-CRP in the MDD group than in the HCs group (*P* < 0.05); however, IL-1β, IL-6, IL-10, and TNF-α did not demonstrate any statistically noteworthy differences between the two groups (*P* > 0.05) (Table 1).

**TABLE 1:** Clinical characteristics and inflammatory parameters.

Variable		MDD (*n* = 66)	HC (*n* = 43)	*t*/*x*^2^	*P*-value
Demographic data	Gender (male/female)	27/39	20/23	0.33	0.564^a^
	Age (years)	24.20 ± 9.60	23.67 ± 3.19	1.66	0.099^b^
	Education years	12.30 ± 3.73	16.58 ± 2.42	−6.66	0.001^b^*
	BMI	21.46 ± 4.61	21.81 ± 2.15	−0.40	0.690^b^
	HAMD-17	20.07 ± 4.20	2.31 ± 2.04	20.20	0.001^b^*
Inflammatory factor	Hs-CRP (ng/ml)	116.80 ± 27.35	103.14 ± 24.92	2.38	0.019^b^*
	IL-1β (pg/ml)	195.02 ± 39.58	192.69 ± 40.98	0.27	0.788^b^
	IL-6 (pg/ml)	118.04 ± 26.37	122.74 ± 23.40	−0.86	0.394^b^
	IL-10 (pg/ml)	146.02 ± 35.79	148.67 ± 34.17	−0.35	0.729^b^
	TNF-α (pg/ml)	471.54 ± 123.17	473.89 ± 106.05	−0.08	0.926^b^
CCT	CCT1(s)	55.93 ± 25.87	37.61 ± 11.68	2.70	0.010^b^*
	CCT2(s)	96.62 ± 45.00	68.75 ± 17.45	2.38	0.021^b^*
RBANS	Immediate memory	83.06 ± 17.30	98.00 ± 17.02	−3.06	0.003^b^*
	Visuospatial/constructional	101.47 ± 15.09	105.00 ± 10.84	−0.90	0.371^b^
	Language	88.17 ± 15.95	99.42 ± 10.60	−2.76	0.008^b^*
	Attention	105.39 ± 14.87	115.37 ± 18.25	−2.19	0.033^b^*
	Delayed memory	88.78 ± 14.97	99.37 ± 15.27	−2.48	0.016^b^*
	Total score	467.28 ± 56.46	516.21 ± 44.90	−3.27	0.002^b^*
	Standardized score	90.94 ± 14.06	104.63 ± 12.50	−3.56	0.001^b^*
SCWT	Word time(s)	16.34 ± 4.80	13.24 ± 3.76	2.35	0.023^b^*
	Color time(s)	20.77 ± 4.80	17.44 ± 3.69	2.59	0.012^b^*
	Word-Color_time(s)	16.75 ± 4.82	13.94 ± 5.47	1.94	0.058^b^
	Color-Word time(s)	35.06 ± 9.22	30.06 ± 6.46	2.06	0.044^b^*

*^a^P-value for chi-square test.*

*^b^P-values for two-sample t-test.*

*Significant difference.

*MDD, major depressive disorder; HC, health control; BMI, Body Mass Index; HAMD, Hamilton’s Depression Scale; Hs-CRP, high-sensitivity C-reactive protein, IL-1β, interleukin 1β; IL-6, interleukin 6; IL-10, interleukin 10; TNF-α, tumor necrosis factor α; CTT, Color Trail Test; RBANS, Repeatable Battery for the Assessment of Neuropsychological Status; SCWT, Stroop Color-Word Test.*

### Gut Microbiota Composition Between Major Depressive Disorder Patients and Healthy Control

**FIGURE 1:**
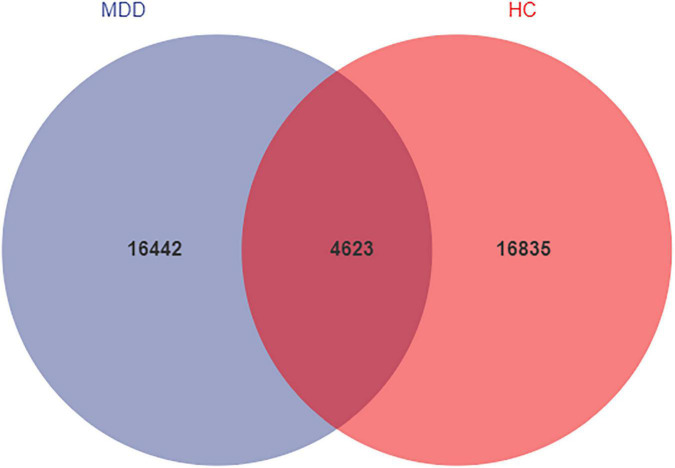
Venn diagram of ASV in MDD and HCs. The blue and red colors represent MDD group and HC group, respectively. The overlapping area between the color blocks indicates the common ASV between two groups, and the number of each block indicates the number of ASV.

We analyzed the microbiomes of 81 total participants (40 MDD and 41 HC) whose stool samples were collected and obtained 5,661,964 high-quality reads across all samples (The raw data is available on NCBI, under the accession number PRJNA776170). These reads were clustered into 37,900 ASVs at 99% sequence similarity. The Venn diagram shows that 4623 ASVs were found in the two groups, while 16,442 and 16,835 ASVs were unique to MDD patients and HCs, respectively (Figure 1). At the level of phylum, these ASVs are mainly concentrated in Firmicutes, Bacteroidetes, Proteobacteria, and Actinobacteria.

**FIGURE 2:**
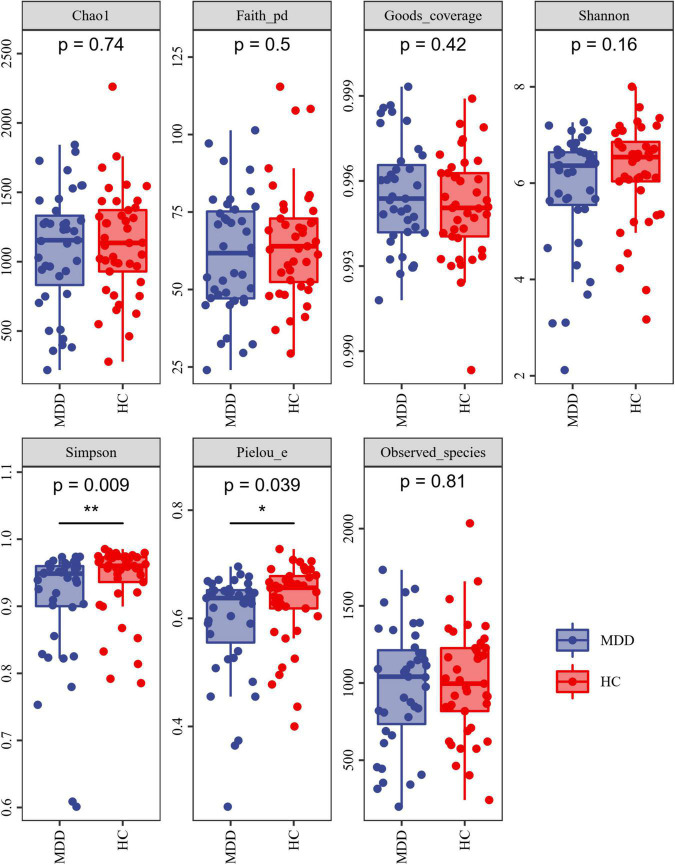
Alpha diversity of MDD and HCs samples. The blue and red colors represent MDD group and HC group, respectively. *P < 0.05 and **P < 0.01.

Alpha-diversity analysis exposed that both the Simpson index and Pielou’s evenness were lower in MDD patients than in HCs (Figure 2). These findings propose that the microbiota of MDD patients is less diverse than that of HCs.

**FIGURE 3:**
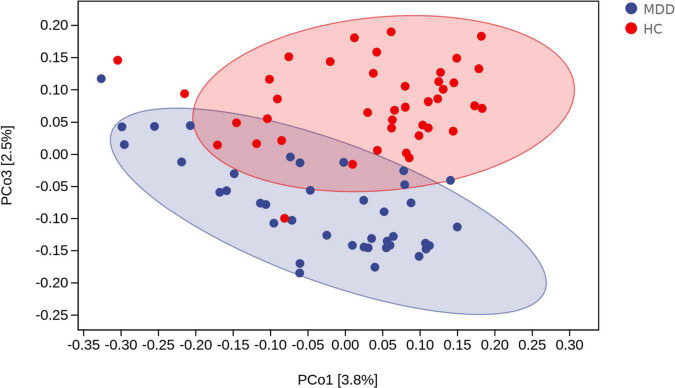
Beta diversity as a principal coordinate analysis (PCoA) plot based on Jaccard dissimilarity. The blue and red colors represent MDD group and HC group, respectively.

Beta diversity analysis uncovered a notable difference in bacterial community composition among MDD subjects and HCs as found by the Jaccard dissimilarity calculations (Figure 3).

**FIGURE 4:**
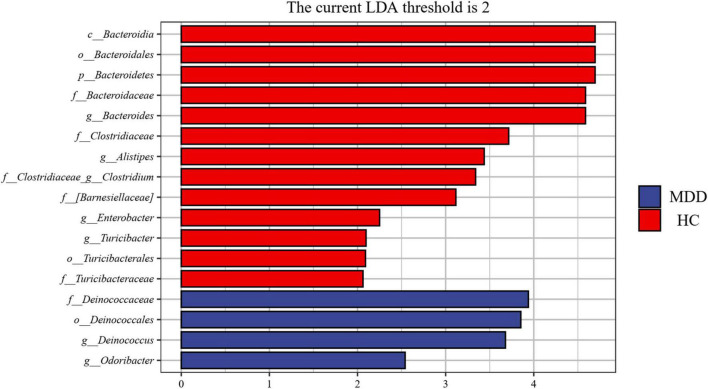
Taxonomic biomarkers found by LEfSe in MDD and HC. Blue and red colors represent MDD and HC enriched family or genus, respectively. Only taxa with *p* < 0.05 and LDA score (log2) are shown.

A linear discriminant analysis (LDA) effect size (LEfSe) test was adopted to investigate the microbiota discrepancies between MDD patients and HCs. At the family level, the relative abundance of *Deinococcaceae* was significantly higher in MDD patients; however, the amount of *Bacteroidaceae*, *Turicibacteraceae*, *Clostridiaceae*, and *Barnesiellaceae* were significantly higher in HCs (Figure 4). At the genus level, the relative abundance of *Deinococcus* and *Odoribacter* was significantly higher in MDD patients; however, the abundance of *Bacteroides*, *Alistipes*, *Turicibacter*, *Clostridium*, *Roseburia*, and *Enterobacter* were much higher in HCs (Figure 4).

**FIGURE 5:**
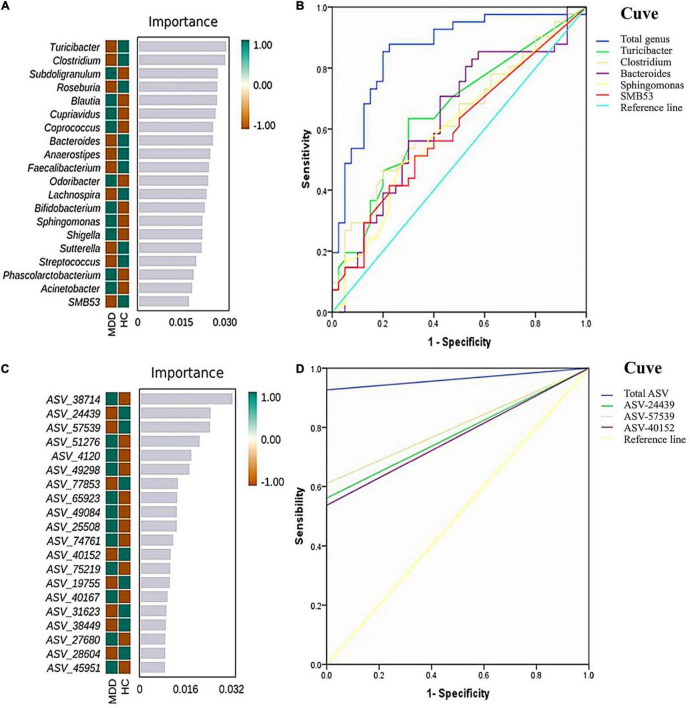
Random forest (RF) classification for gut microbiota in the two groups. The important scores of the confirmed attributes are presented at the genus level **(A)** and ASVs level **(C)**, respectively. The receiver operating characteristic (ROC) curves were made by single and combined (total genus/ASVs) microorganism at the genus level **(B)** and ASVs level **(D)**, respectively.

To determine the biomarkers for discriminating between MDD patients and HCs at the genus level, we applied a RF and Boruta machine-learning algorithm, which found 20 important genera. We then executed a stepwise regression analysis based on the relative abundance of altered genus. This examination revealed that the most pronounced discrepancies between MDD and HC subjects were due to the relative abundance of *Turicibacter*, *Clostridium*, *Bacteroides*, *Sphingomonas*, and *SMB53*. The ROC analysis showed that the AUC was 0.656, 0.633, 0.635, 0.601, and 0.596, separately. When combining these indicators, the AUC was 0.859. At the ASV level, and while employing the same method, we found the most significant discrepancies between MDD and HC subjects were due to the relative abundance of ASV-24439 (*Faecalibacterium prausnitzii*), ASV-40152 (*unidentified_Bacteroides*) and ASV-57529 (*unidentified_Roseburia*). The ROC analysis showed that the AUC was 0.780, 0.805, and 0.768, separately. When combining the three indicators, the AUC was 0.963 (Figure 5).

### Relationship Between Microbial Biomarkers and Inflammatory Parameters, Depressive Symptoms, and Cognitive Function

**FIGURE 6:**
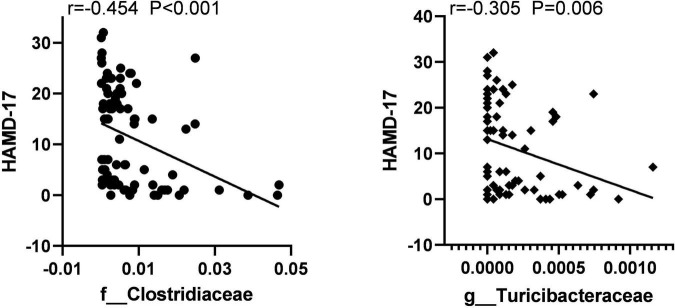
The scatter plot of correlation between Clostridiaceae, Turicibacteraceae abundance, and the HAMD-17 scores in two groups.

**FIGURE 7:**
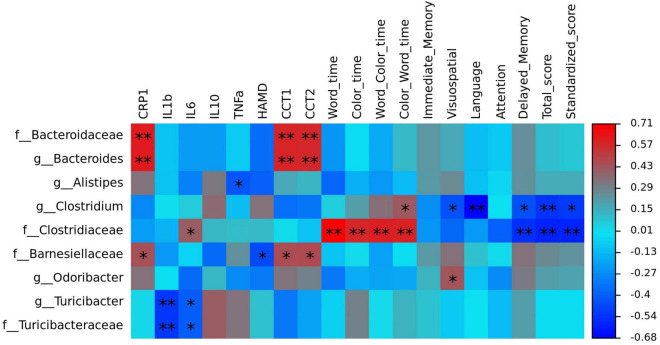
The heatmaps of spearman correlation coefficient matrix between significant different gut bacteria and inflammatory factors, cognitive function. Red indicates positive correlations, while blue indicates negative. *P < 0.05 and **P < 0.01. Note: HAMD, Hamilton’s Depression Scale; CRP, C-reactive protein, IL-1β, interleukin 1β; IL-6, interleukin 6; IL-10, interleukin 10; TNF-α, tumor necrosis factor α; CTT, Color Trail Test.

To identify the key microbiota, we performed correlation analysis and found that relative abundance of *Clostridiaceae* and *Turicibacter* were negatively correlated with the total score HAMD-17 in all subjects (Figure 6). In MDD patients, the relative abundance of *Bacteroidaceae* and *Bacteroides* were both positively correlated with hsCRP and cognitive function, such as CCT1 and CCT2. The relative abundance of *Alistipes* was positively correlated with IL-6, Word time, Color time, Word-Color time, and Color-Word time, and negatively correlated with Delayed Memory, Total score, and Standardized score. The relative abundance of *Deinococcus* was positively correlated with the Color-Word time, and negatively correlated with Visuospatial/Constructional, Language, Delayed Memory, Total score, and Standardized score in RBANS. The relative abundance of *Turicibacteraceae* and *Turicibacter* were both negatively correlated with IL-1β and IL-6 (Figure 7).

## Discussion

In our examination, we found a significant cognitive decline in MDD patients compared to healthy subjects. Our findings support the findings of previous research. MDD patients more commonly demonstrate cognitive disturbances than the general population (Pan et al., 2019). Although a disturbance of mood is the traditional definition of MDD, impaired cognitive function is often considered a result of the disorder (Austin et al., 2001). We define cognitive function as the mental processes of receiving, using, and preserving information, and these are sorted into fields like attention and memory (Ahern and Semkovska, 2017). Many researchers have also uncovered cognitive deficits in processing speed, attention, learning abilities, long-term memory, autobiographical memory, and executive function. For example, a meta-analysis of 24 studies, one comprised of 784 MDD patients, quantified moderate cognitive deficits in executive function, memory, and attention of MDD patients relative to HCs (Snyder, 2013; Rock et al., 2014). The state of knowledge at the moment proposes that cognitive impairment in MDD patients is an important determining factor of a patient’s individual functional outcomes. Cognitive impairment has been observed to remain present after MDD remission, to decline with recurrent depressive episodes, and to serve as an important predictor of relapse (Reppermund et al., 2009). Furthermore, previous research has proposed that cognitive remission should serve as a therapeutic target for restoring, functioning, and preventing relapse (Bortolato et al., 2016).

Previous studies have identified depression as linked to differences in the gut microbiota of patients, and our study contributes to this body of work (Kelly et al., 2016; Huang et al., 2018; Liśkiewicz et al., 2021; Yuan et al., 2021). Here, we found Alpha-diversity and Beta-diversity were different between MDD subjects and HCs. However, a number of studies have also reported different conclusions (Naseribafrouei et al., 2014; Jiang et al., 2015).

We discovered that gut microbiota was significantly different between MDD and HCs at the family level and genus level.

Some of our results were congruent with outcomes of previous studies. Zhang et al. (2021) found decreasing *Clostridiaceae* and *Alistipes* in MDD, and that the severity of depression correlated with bacterial composition. Zheng et al. (2016) found that the families *Bacteroidaceae and Lachnospiraceae* and the genera *Alistipes* and *Clostridium XlVa* were overrepresented in HCs. Another study noted that the overall content of Firmicutes was dramatically decreased in the MDD group than in the HC group; at the family level, *Clostridiaceae* were decreased; and at the genus level, *Clostridium* and *Alistipes* were decreased (Huang et al., 2018). However, contrary to our findings, some studies showed that the relative proportions of *Bacteroides*, *Turicibacter*, and *Clostridium* were increased in the depressed group, whereas *Dialister* was decreased (Jiang et al., 2015; Kelly et al., 2016; Liu et al., 2020).

Correlation analysis showed that *Clostridiaceae* and *Turicibacter* were negatively correlated with the total HAMD-17 score in all subjects. In MDD patients, *Bacteroidaceae* and *Bacteroides* were both positively correlated with hsCRP, CCT1, and CCT2. *Alistipes* was positively correlated with IL-6, Word time, Color time, Word-Color time, and Color-Word time, and negatively correlated with Delayed Memory, Total score, and Standardized score. *Deinococcus* was positively correlated with the Color-Word time and negatively correlated with Visuospatial/Constructional, Language, Delayed Memory, Total score, and Standardized score in RBANS. *Turicibacteraceae* and *Turicibacter* were both negatively correlated with IL-1βand IL-6.

Currently, studies on the correlation between cognitive impairment and gut microbes have focused on AD. Many studies have found that neuroinflammation plays an important role in cognitive impairment. Gut microbiota could affect intestinal permeability and trigger systemic proinflammatory cytokines. Systemic inflammation could accelerate cognitive impairment by acting synergistically (Calsolaro and Edison, 2016; Li et al., 2019). *Bacteroides* are commensal and Gram-negative, and are some of the most copious bacteria present in the human gastrointestinal system. Certain strains of the Bacteroidetes species are considered generally advantageous to human health because of their many abilities (Sears, 2009). However, when people experience stress such as shock, abuse, the loss of a family member, etc., certain strains of Bacteroidetes species such as *Bacteroides fragilis* can secrete lipopolysaccharide (LPS), bacterial amyloids, endotoxins (such as fragilysin) and exotoxins. These neurotoxins stimulates the release of TNF-α, IL-1β, IL-8, gamma interferon (IFN-γ), CXC ligand 8 (CXCL8) and other inflammatory cytokines and chemokines in various cell types, leading to inflammatory response toward these bacterial molecular pathogens (Lukiw, 2016; Alexandrov et al., 2020). Activated inflammatory responses can disrupt both the intestinal mucosal barrier as well as the BBB, and further, they can activate the microglia of CNS (Alexandrov et al., 2020). Activated microglia were found to participate in the secretion of pro-inflammatory cytokines, including IL-1β, IL-6, TNF-α, and TGF-β, thus aiding the development of cognitive impairment in individuals with neurological disorders (Giau et al., 2018). *Alistipes* is also pro-inflammatory bacteria. Hennezel et al. (2017) revealed that the levels of *Alistipes putredinis* and *Alistipes finegoldii* possess a moderate-to-strong correlation with IL-6, TNF-α and IL-1 production. Another study showed that the *Alistipes* genus participated in prompting inflammation and tumorigenesis in an IL-6-dependent manner (Moschen et al., 2016). However, there was no significant correlation between *Alistipes* and IL-1, IL-6 in this study, and a negative correlation between *Alistipes* and TNF-α. Therefore, *Alistipes* can aggravate cognitive impairment by other ways than inflammatory response.

We found that *Clostridiaceae* and *Turicibacter* were both negatively correlated with IL-1β, IL-6, TNF-α, and HAMD-17 scores. These bacteria participate in the production of SCFAs such as butyrate, acetic acid, and valeric acid. SCFAs demonstrate anti-inflammatory functions, regulate the differentiation of T-lymphocytes, function as energy sources for the intestinal epithelium, and act as a key regulator for proper intestinal permeability (Haghikia et al., 2015). Furthermore, butyrate can cross the BBB and disrupt the function of the hippocampus, in addition to promoting BDNF expression, which has previously been found to cause antidepressant-like effects in animal models (Soret et al., 2010). Consequently, insufficient butyrate-producing bacteria in MDD subjects may influence MDD’s pathology.

In conclusion, this study found that some increased pro-inflammatory bacteria and some decreased anti-inflammatory bacteria in MDD patients are associated with inflammatory factors and cognitive function. Gut microbiota may be the cause of cognitive impairment in patients with MDD. Environmental and genetic factors cause changes in gut microbiota, and altered gut microbiota promotes inflammatory responses in the intestinal, peripheral blood and CNS, which in turn leads to cognitive impairment. However, an altered gut microbiota can also be a result of depressive symptoms and impaired cognitive function. Changes to lifestyle, eating habits, and behavior that may cause dysbiosis of gut microbes can accompany/precede diagnosis of MDD. One limitation of this study is that we conducted only correlation analyses, thus is difficult to determine the causal relationship between gut microbes and cognitive impairment, which would require to be verified through animal experiments. In addition, an inflammation response may be one of the mechanisms of the interaction between gut microbiota and the CNS. The imbalance of neuroendocrine and metabolism should also be a concern.

Our study submits a prediction model for microbial biomarkers at the genus and ASV levels, which showed high accuracy (AUC = 0.859 and 0.963) signifying an effective classification model. According to previous studies, gut microbes can be used as biomarkers for distinguishing MDD patients form HCs. Lai et al. (2021) created an RF classification model based on genus levels with an AUC of 0.890. Further, their RF classification model based on species levels attained a higher AUC than their genus level model, achieving 0.997. It is commonly accepted that AUC scores can be taken as a ‘single number’ measure to evaluate and compare classifiers, where scores of <0.70 are considered poor, 0.70–0.79 are considered fair, 0.80–0.89 are considered good, and 0.90–1.00 are considered excellent (Cicchetti, 2001). The AUC score of the model used here was calculated as 0.86 and 0.96, thereby indicating good discrimination and robustness.

Compared with previous studies, there are many inconsistent results. The heterogeneity of the microbiome research is understood to be connected to various factors that been stated in limitation.

Our study has certain limitations. One limitation is that we used a sample size that was too small to eliminate the chance of false positives. Second, we applied 16S rRNA sequencing, which only detects taxa at the genus level. Therefore, there may have been key associations at the species or strain levels that were overlooked. Third, dietary structure is understood to significantly impact the composition and function of the gut microbiota. In the present study, we did not collect complete dietary data, which could have biased our results. Fourth, geographical distribution may be a potential confounder, which could affect the composition of gut microbiota. The generalization of our results needs additional corroboration in more regions of China, and in other countries, as well.

## Conclusion

This study demonstrates that compared to HCs, MDD patients showed impaired cognitive function and had a significant abnormal gut microbiota composition, and increased CRP. Altered gut microbiota is closely associated with inflammatory factors and cognitive function in MDD patients.

## Data Availability Statement

The original contributions presented in the study are publicly available. This data can be found here: https://www.ncbi.nlm.nih.gov/search/all/?term=PRJNA776170.

## Ethics Statement

The studies involving human participants were reviewed and approved by Ethical Committee of the First Hospital of Shanxi Medical University. The patients/participants provided their written informed consent to participate in this study. Written informed consent was obtained from the individual(s) for the publication of any potentially identifiable images or data included in this article.

## Author Contributions

KZ and NS designed the experiments. MG, YZ, HT, LL, PW, AZ, CY, and ZL participated in the collection and analysis of clinical data, stool samples, and blood samples of all subjects. PL analyzed data and wrote the manuscript. All authors contributed to the clinical data collection and assessment.

## Conflict of Interest

The authors declare that the research was conducted in the absence of any commercial or financial relationships that could be construed as a potential conflict of interest.

## Publisher’s Note

All claims expressed in this article are solely those of the authors and do not necessarily represent those of their affiliated organizations, or those of the publisher, the editors and the reviewers. Any product that may be evaluated in this article, or claim that may be made by its manufacturer, is not guaranteed or endorsed by the publisher.

## Footnotes

1
https://www.genescloud.cn/chart/CorHeatmap
